# Orodispersible Polymer Films with the Poorly Water-Soluble Drug, Olanzapine: Hot-Melt Pneumatic Extrusion for Single-Process 3D Printing

**DOI:** 10.3390/pharmaceutics12080692

**Published:** 2020-07-22

**Authors:** Hui-Won Cho, Seung-Hoon Baek, Beom-Jin Lee, Hyo-Eon Jin

**Affiliations:** 1 College of Pharmacy, Ajou University, Suwon 16499, Korea; huiwon825@ajou.ac.kr (H.-W.C.); shbaek@ajou.ac.kr (S.-H.B.); bjl@ajou.ac.kr (B.-J.L.); 2 Research Institute of Pharmaceutical Science and Technology, Ajou University, Suwon 16499, Korea

**Keywords:** three-dimensional printing technology, orodispersible film, disintegration, dissolution, hot-melt extrusion, poorly water-soluble drugs

## Abstract

Amorphous solid dispersions (ASDs) improve the oral delivery of poorly water-soluble drugs. ASDs of olanzapine (OLZ), which have a high melting point and low solubility, are performed using a complicated process. Three-dimensional (3D) printing based on hot-melt pneumatic extrusion (HMPE) is a simplified method for producing ASDs. Unlike general 3D printing, printlet extrusion is possible without the preparation of drug-loaded filaments. By heating powder blends, direct fused deposition modeling (FDM) printing through a nozzle is possible, and this step produces ASDs of drugs. In this study, we developed orodispersible films (ODFs) loaded with OLZ as a poorly water-soluble drug. Various ratios of film-forming polymers and plasticizers were investigated to enhance the printability and optimize the printing temperature. Scanning electron microscopy (SEM) showed the surface morphology of the film for the optimization of the polymer carrier ratios. Differential scanning calorimetry (DSC) was used to evaluate thermal properties. Powder X-ray diffraction (PXRD) confirmed the physical form of the drug during printing. The 3D printed ODF formulations successfully loaded ASDs of OLZ using HMPE. Our ODFs showed fast disintegration patterns within 22 s, and rapidly dissolved and reached up to 88% dissolution within 5 min in the dissolution test. ODFs fabricated using HMPE in a single process of 3D printing increased the dissolution rates of the poorly water-soluble drug, which could be a suitable formulation for fast drug absorption. Moreover, this new technology showed prompt fabrication feasibility of various formulations and ASD formation of poorly water-soluble drugs as a single process. The immediate dissolution within a few minutes of ODFs with OLZ, an atypical antipsychotic, is preferred for drug compliance and administration convenience.

## 1. Introduction

The active pharmaceutical ingredients (APIs) corresponding to class II of the Biopharmaceutical Classification System (BCS) comprise more than 50% of all ingredients. They are characterized by their low water solubility resulting in poor oral bioavailability [1]. Improving the solubility of drugs is a significant challenge in the drug discovery field. Strategies have many approaches, such as micronization [2], cocrystallization [3], complexation [4], nanoparticle engineering, and solid dispersion [5,6,7,8]. Amorphous solid dispersions (ASDs) are dispersed amorphous drugs in a suitable polymeric carrier matrix, which typically form amorphous APIs leading to improved water solubility and dissolution rates due to the amorphous state exhibiting increased free energy, enthalpy, and entropy compared with the crystalline form. ASDs are characterized by lower cost for preparations, simple formulation, and ease of scale-up [9]. This technology has received much attention in the pharmaceutical industry over the past 20 years, and commercial products have been developed [10].

Olanzapine (OLZ) is an atypical antipsychotic used for the treatment of schizophrenia and other mental illnesses due to its high efficiency and minimal side effects [11]. Various studies have investigated the enhanced bioavailability of drugs such as OLZ belonging to BCS class II poorly water-soluble drugs using solid dispersions [11,12]. OLZ is commercially available as orally disintegrating tablets (ODTs) such as Zyprexa^®^ [13]. The dosage forms for oral disintegration have been evaluated as an effective alternative treatment for poorly compliant patients with schizophrenia for decreasing psychopathology and providing improved drug compliance capacity [14].

As an oral dosage form, orodispersible films (ODFs) in a form similar to ODTs are quickly dissolved by saliva on the tongue, obviating the need to swallow the drug, unlike normal oral pathways. ODFs are a particularly preferred formulation form for patients who have difficulty swallowing tablets and can be taken without water. ODTs are usually produced using lyophilization, which accelerates disintegration and results in a fragile form, which induces manufacturing, storage, handling, and management problems. Conversely, ODFs have excellent mechanical strength and flexibility, resulting in lower production costs than lyophilization [15]. The solvent-casting method is also used, offering the advantage of the ease of production and low system set-up costs for one ODF method, but faces the inherent and inevitable problems of preparation, such as high batch-to-batch variability and the existence of bubbles and residual solvents in the films [16]. As more effective methods are required to overcome these limitations, hot-melt extrusion (HME) is a relatively simple process, and the solvent-free approach has been applied to produce ODFs in recent years.

HME has been included for blending and heating materials through one or more rotating screws before the manufacturing of filaments, granules, or powders via extrusions [16,17,18,19,20,21]. In the process, establishing appropriate extrusion parameters is affected by the formulation and physicochemical properties of the API and polymers, including glass transition and melting temperatures, and the miscibility or solubility of the API in the polymer carrier. The polymer carrier composed within the formulation has a limited number of options for approved pharmaceutical uses, and its properties have the greatest influence in determining processing conditions [10]. HME use in pharmaceutical fields offers several advantages, such as no requirement for the compressibility of APIs, better content uniformity in extrudates, and usability for the formulation of various thin films. Moreover, the preparation of a solid molecular dispersion of APIs into different hydrophilic polymer matrices is available to improve the solubility and dissolution rate [17,22]. The APIs are melted or solubilized in the polymeric carriers, and rapid cooling and high viscosity lead to thermodynamically stabilized ASDs [23]. Therefore, HME was used as a step to prepare ASDs of poorly water-soluble drugs for ODF production with increased dissolution characteristics, and polymer materials and various processing parameters according to the formulation and selected methods were evaluated.

Three-dimensional (3D) printing technology forms objects by additive manufacturing depending on the information in the files sent to the software program. Different from administration in the form of fixed doses in large manufacturing, it enables the flexible production of patient-tailored drugs to meet unsatisfied clinical needs. 3D printing technologies, such as inkjet, fused deposition modeling (FDM), and selective laser sintering (SLS), are more suitable for wide applications in manufacturing pharmaceutical dosage forms [24]. Among the many technologies applicable to 3D printing, the most actively used technology in the pharmaceutical industry is material extrusion, which is described as an FDM [25,26,27]. FDM 3D printing is typically explained in two steps: (i) mixing and heating materials using HME screws to obtain filaments and (ii) subsequent manufacture of objects by melting the prepared filaments. These multi-step processes have a significant limitation in FDM 3D printing. Hot-melt pneumatic extrusion (HMPE) for single-step 3D printing as different forms of HME was used to eliminate intermediate steps, having faster possibilities for the production of substances. Powders or pellets that are directly fed into the HMPE system can be a simplified FDM paradigm, which is tailored to the printing process, the release of the drug, and dosage form [16,25,26,28].

**Figure 1:**
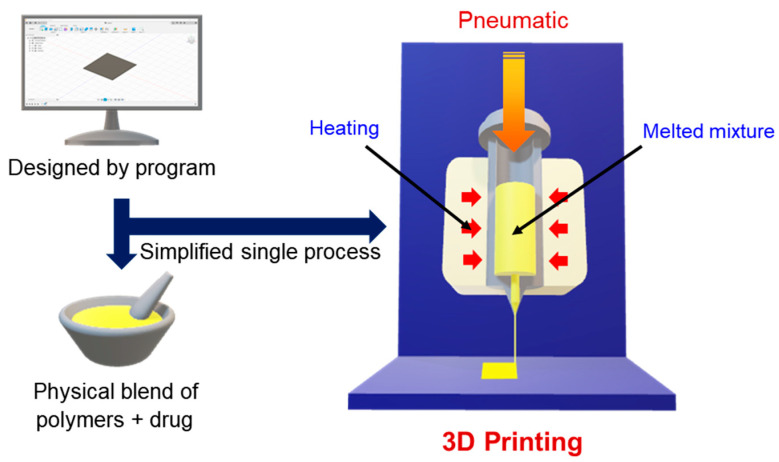
Experimental schematic of the three-dimensional (3D) printing process using hot-melt pneumatic extrusion (HMPE).

In this study, we developed ODFs loaded with ASDs of a poorly water-soluble drug, OLZ, in hydrophilic polymeric carriers, and used the direct powder extrusion of single-step 3D printing as an innovative tool (Figure 1). Diverse parameters and surface morphology were investigated for stable film printing capacity. The formulations were evaluated by disintegration time and the enhanced dissolution rate of ODFs.

## 2. Materials and Methods

### 2.1. Materials

Olanzapine was provided by Dong-A Pharm Co., Ltd. (Gyeonggi, Korea). Polyethylene oxide (POLYOX WSR N10 LEO) 100,000 was provided from Colorcon Ltd. (Dartford, UK). Kollidon^®^ VA 64, poloxamer 407 (Kolliphor^®^ P407), and poloxamer 188 (Kolliphor^®^ P188) were donated by BASF (Ludwigshafen, Germany). All other chemicals were of analytical grade.

### 2.2. Methods

#### 2.2.1. Design of 3D Printing Films

Autodesk Fusion 360 software (ver. 2.0.7813, Autodesk, Inc., San Rafael, CA, USA) was used to design the 3D model. For compatibility with a 3D printer (ROKIT INVIVO Corp., Seoul, Korea), 3D structure files were converted to G-Code in slicer settings by NewCreatorK software (ver. 1.57.76, ROKIT INVIVO Corp., Seoul, Korea). Many parameters were screened to achieve good quality printlets (Table 1). The printing and moving speeds available in the software were evaluated and various conditions were optimized for use in printing formulations. The investigated film size was 20 × 25 mm in 2 layers, including 5 mg of OLZ fabricated by adding a layer drawn through the nozzle in additive manufacturing. The printing process was carried out for approximately 30 min. Figure 1 shows the experimental schematic and the overall process of 3D printing.

#### 2.2.2. Preparation of Hot-Melt Pneumatic Extrusion (HMPE)

**Figure 2:**
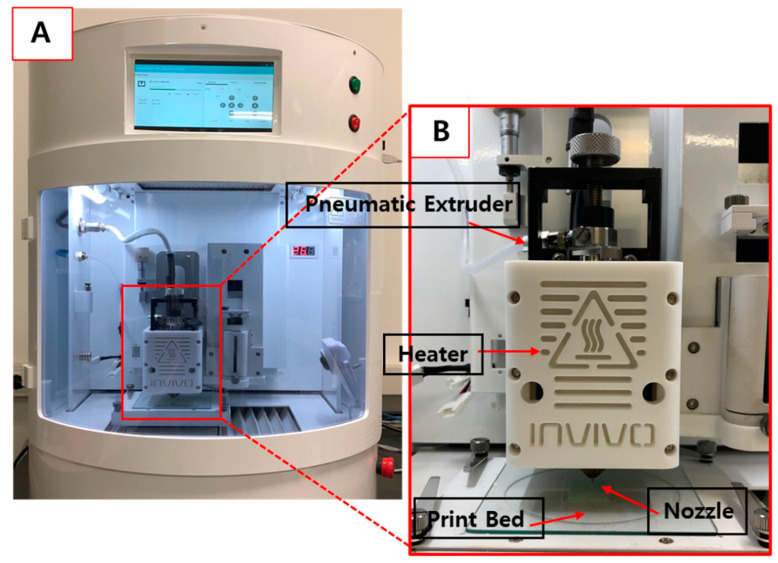
Images of (**A**) the 3D printer with combined HMPE; (**B**) the process by which objects are printed through nozzles on the print bed.

Various ratios of polymeric carrier matrices were weighed and mixed using a mortar and pestle blender (Samhwa Ceramic Co., Seoul, Korea), and additional mixing was performed via a blender. Each mixture was prepared at 5 g, filled into the printing barrel, and heated. The 3D printer using HMPE printed the printlets that were prepared with various ratios of polymeric carrier matrices with a nozzle diameter of 0.4 mm (Figure 2). The print bed moved to produce ODFs. We optimized the major parameters, including printing temperature and air pressure, by changing the conditions. The extrusion temperature used in each formulation was optimized by increasing the temperature by 5–10 °C, starting at 100 °C. Pneumatic pressure was first assessed at 200 kPa and then increased by 50 kPa. It was appropriate for all formulations when a value between 450 kPa and 500 kPa was used.

#### 2.2.3. Film Thickness and Mechanical Properties

The mechanical properties of the films were measured using an H5KT texture analyzer (Tinius Olsen Corp., Horsham, PA, USA) with 500 N-rod cells. The prepared films were tested at a controlled crosshead speed of 0.04 inches/min. The film samples were evaluated and recorded until they broke. The thickness of the film was employed using a constant pressure thickness measuring instrument PG-12J (TECLOCK, Nagoya, Japan) at various sections, and the average was recorded.

#### 2.2.4. Drug Content

A film (4 cm^2^ pieces including 5 mg OLZ) of each formulation was placed in 100 mL of 20 mM phosphate buffer (pH 6.8) and sonicated when the film was sufficiently dissolved (n = 3). Then, the solutions were filtered through a 0.45 µm syringe filter. Samples were diluted in a 25 mL volumetric flask, and the absorbance was analyzed at 273 nm using high-performance liquid chromatography (HPLC, Waters^®^ 2695, Waters Corp., Milford, MA, USA).

#### 2.2.5. Disintegration Test

Disintegration tests of the films were performed using a pharmacopeial disintegration apparatus type A, namely the DIT-200 disintegration apparatus (Labfine Inc., Seoul, Korea) [19]. Films were mounted in the disintegration vessel, and a 900 mL amount of deionized water was used as the disintegration medium at 37 °C. The time was recorded when the films were disintegrated, and tests were conducted three times.

#### 2.2.6. High-Performance Liquid Chromatography (HPLC) Analysis

The concentrations of OLZ were analyzed using a Waters^®^ 2695 HPLC system (Waters Corp.). A 5 μm XTerra^®^ reverse phase C18 (250 × 4.6 mm) column (Waters Corp., Milford, MA, USA) was used with an isocratic elution method that contained a mobile phase of 12.86 mM potassium dihydrogen phosphate buffer (pH 6)/acetonitrile (60:40 *v/v*) [29]. A flow rate of 1 mL/min with UV detection at 258 nm was set. The injection volume was 20 μL. The method showed a liner correlation between drug concentration from 0.5 µg/mL to 50 µg/mL. All HPLC data assays were performed in triplicate (n = 3).

#### 2.2.7. Scanning Electron Microscope (SEM)

The surface morphology of the ODFs was assessed using field-emission scanning electron microscopy (FE-SEM, JSM-6700F, JEOL, Akishima, Japan). Samples were coated with platinum under vacuum for 80 s. Imaging was performed at various magnifications operating at a voltage of 5 kV.

#### 2.2.8. Differential Scanning Calorimetry (DSC)

Sample thermal patterns were analyzed using a DSC instrument (DSC 200 F3 Maia, NETZSCH, Selb, Germany). The device was calibrated using indium. Samples were prepared in a sealed aluminum pan, which was set from 5 °C to 230 °C at a heating rate of 10 °C/min. Pure nitrogen gas was streamed at a flow rate of 50 mL/min in all the DSC analyses. The melting and transition points were obtained from the heat flow data.

#### 2.2.9. Powder X-ray Diffraction (PXRD)

The crystallinity of each powder, powder mixture, and 3D printed samples were examined using a powder X-ray diffractometer (D/max-2500V/PC, Rigaku, Tokyo, Japan). PXRD studies were performed with Cu-K radiation at 40 kV and 150 mA, and the sample was scanned by adding 0.02° from 5° to 50° at a speed of 1 s/scan. The presence of peaks was considered to be the crystalline form of the materials.

#### 2.2.10. In Vitro Dissolution Test

An in vitro dissolution test was performed using a United States Pharmacopeia (USP) Apparatus II paddle method (LOGAN Instruments Corp., Somerset, NJ, USA). The following simulated salivary medium was prepared for this study: KH_2_PO_4_ (12 mM), NaCl (40 mM), and CaCl_2_ (1.5 mM) adjusted to pH 7.4 using NaOH. The in vitro evaluation was performed using 900 mL of a dissolution fluid with a paddle rotation speed of 100 rpm and 37 ± 0.5 °C [30,31]. At time intervals of 1, 3, 5, 7, 10, 15, 20, 30, 45, and 60 min, 5 mL of the sample solution was withdrawn from the medium and filtered through a syringe filter (0.45 µm). The filtrates were assayed using HPLC under the described conditions. All dissolution tests were performed under sink conditions. The dissolution profiles were plotted as the average of the OLZ concentrations repeated three times.

#### 2.2.11. Statistical Analysis

Data were expressed as mean ± SD of three independent experiments. Comparison among multiple groups was performed using one-way ANOVA with the Tukey–Kramer test. *p* < 0.05 was considered to be the minimal level of significance.

## 3. Results and Discussion

### 3.1. Preparation and Evaluation of ODFs for 3D Printing

#### 3.1.1. Optimization of ODF Printing Parameters

**Figure 3:**
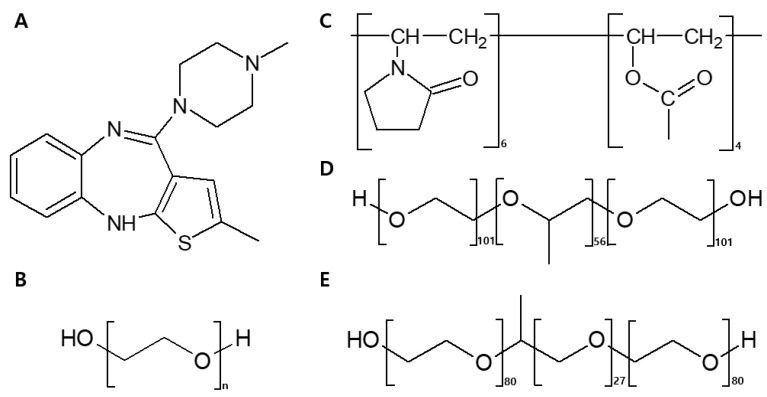
Chemical structures of polymers and drugs used in this study: (**A**) olanzapine (OLZ), (**B**) polyethylene oxide (PEO), (**C**) Kollidon^®^ VA64 (PVPVA64), (**D**) poloxamer 407 (P407), and (**E**) poloxamer 188 (P188).

For pneumatic extrusion using a 3D printer, suitable materials were selected (Figure 3). Polyethylene oxide (PEO) has a range of molecular weights, of which N-10 (molecular weight about 100,000 Da, Dow Chemicals) was the most appropriate film-forming polymer for ODFs. In addition, it provided mechanical flexibility favorable to FDM 3D printing [32,33]. Kollidon^®^ VA64 (PVPVA64), which is often used to prepare ASDs using HME, has been shown to have good miscibility with OLZ and achieve instant release [34,35]. Poloxamer 407 (P407) and poloxamer 188 (P188) are ABA-type tri-block copolymers, containing approximately 70%/30% and 80%/20% of PEO/poly(oxypropylene) (PPO). These are used to prepare solid dispersions, and to enhance the bioavailability of low-solubility drugs in oral solid dosage forms [36]. Therefore, we added P407 and P188 as plasticizers to improve the dissolution and disintegration rates of ODFs. The chemical structures of OLZ, PEO, PVPVA64, P407, and P188 are shown in Figure 3.

In the 3D printer, the powder blend was filled into the extruder barrel and designed in the direction of a vertical orientation, allowing the powder to facilitate the pneumatic extrusion and minimize the presence of air bubbles in the materials. This method was possible to reduce the waste compared to conventional technologies combined with HME and FDM printing. A 3D printer with direct powder extrusion is also a simple and economical process because it does not require complicated steps and materials [26]. Excipients composed of polymeric carriers were selected in consideration of suitable materials for pneumatic extrusion. The formulation was chosen to design films with 20 × 25 mm diameter with the final 100 mg of ODFs. Preliminary printing parameters with formulations are listed in Table 1. The suitability of printing was assessed by its excellent finish quality and smooth surface formation, and the moving and printing speeds were adjusted to those that satisfy the factors and lead to a rapid process [37]. During additive manufacturing, the bed temperature prevented printing from being pushed and helped maintain a sturdy shape.

#### 3.1.2. Optimization of Polymeric Carrier Composition for ODF Printing

Many factors, including the optimization of the printing temperature and extrusion capacity, should be evaluated as necessary for 3D printing. Materials prepared for 3D printing should not be brittle or too soft to be extruded from the nozzle [38]. Therefore, we tested the placebo (P) samples without the drug to determine the printing temperature and extrusion capability of the polymers (Table 2). Printed mixtures from P1 to P4 showed good extrusion and film-forming capacity. The film-forming capacity began to destabilize in P5, and it was not extruded in P6 when it contained more than 50% of PVPVA64 [39].

The PEO single polymer was extruded at 100 °C and allowed a single pneumatic extrusion without any other excipients (P1 in Table 2). However, when the PEO was mixed with 5% OLZ (F1 in Table 3), the printing temperature of ODF was approximately 170 °C owing to the effect of OLZ with a high melting point [40]. A high printing temperature was not appropriate because the loaded material burned quickly before printing. PVPVA64 was added to solve this problem because the presence of PVPVA64 enabled milder printing conditions such as lower temperatures [25]. The ratios of PEO/PVPVA64 were evaluated by increasing the amount of PVPVA64, and formulations from 10% to 30% of PVPVA64 were able to print (F2–4 in Table 3). The printing temperature was decreased by adding PVPVA64, but there was no difference in the printing temperature of F3 (20% PVPVA64) and F4 (30% PVPVA64) in Table 3. They had the same printing temperature when formulations contained over 20% of PVPVA64. However, when more than 40% of PVPVA64 was included, the extrusion became more brittle and less extrudable over time (data not shown).

#### 3.1.3. Surface Morphology of ODFs with Polymeric Carriers using only SEM

**Figure 4:**
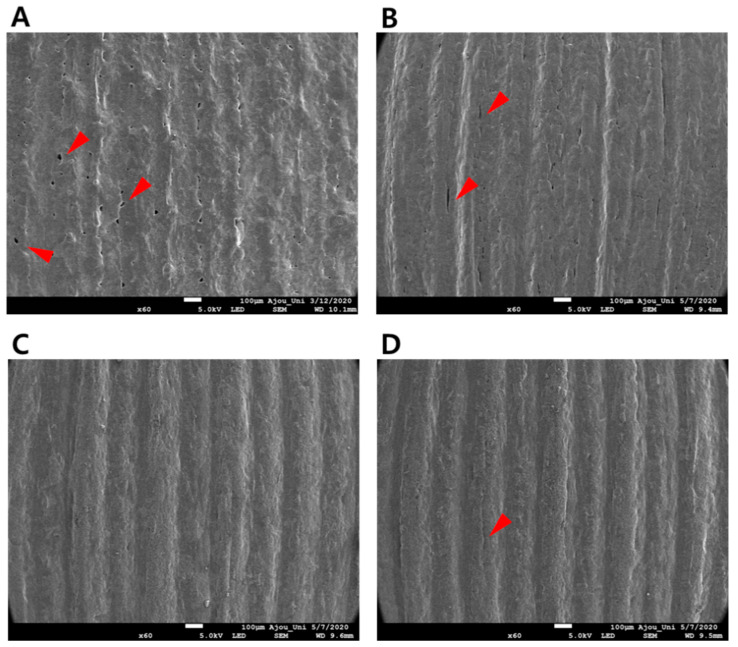
Scanning electron microscopy (SEM) images of the 3D printed ODFs of (**A**) F1, (**B**) F2, (**C**) F3, and (**D**) F4.

The SEM images of ODFs from F1 to F4 in Table 3 are shown in Figure 4. The formulation containing only the PEO polymer with OLZ showed porous sections and position cutoff, resulting in roughened surfaces (Figure 4A). The 10% PVPVA64 also had porous parts (Figure 4B). The films showed a smoother surface with fewer pores when PVPVA64 was more than 20% in F3 and F4 (Figure 4C,D). Increased ratios of PVPVA64 affected the film-forming capacity, such as surface morphology. The degree of incorporation between the polymers and the drug was improved by the addition of PVPVA64, acting as a binding agent. Although F3-ODF and F4-ODF showed similar surface morphology under SEM, F4-ODF had a small crack. Excessive addition of PVPVA64 decreased the flexibility of the films so that the polymeric carrier composed of 75% PEO and 20% PVPVA64 was selected for subsequent studies.

### 3.2. Final Formulations and Characterization of ODFs

Bhyan et al. reported that plasticizers can influence multiple elements, such as enhanced mechanical properties and fast dissolution patterns, and the absorption rate of drugs was improved by the use of a plasticizer [41]. P407 and P188 have been widely used as plasticizers in hot-melt extrusion technology [34,42]. Therefore, we added different types of poloxamer to F3 while maintaining the 20% PVPVA64. The compositions of formulations with a poloxamer, P407 or P188, are shown as F5 and F6, respectively, in Table 3. The effects of the plasticizer on the thickness, strength, and disintegration rate were assessed, as shown in Table 4.

The printed 20 × 25 mm ODFs were shown to fit a total mass of 100 mg, which was designed to contain a 5 mg dose of OLZ. The drug content in ODFs was measured using HPLC analysis and the theoretical drug content in all final formulations, i.e., F3, F5, and F6 (Table 4). Assay results indicated that the drug was not degraded during the printing process. While ODFs made by 3D printing had the exact size of the X and Y axes, the consistency of thickness of the z-axis was particularly important in the manufacturing process because thickness affects the total amount of printlets directly related to the drug’s content. All three formulations, F3, F5, and F6, showed definite thickness ranges in ODFs (Table 4).

The mechanical properties of ODFs were tested because ODFs require suitable strength for handling and transport. P407 and P188 were used as plasticizers. In comparison to F3-ODF, the strength of F5-ODF with P407 decreased, whereas the strength of F6-ODF with P188 was slightly increased (Table 4).

The disintegration test method prescribed in a pharmacopoeia, the Petri dish method, and the slide-frame method have been used as three types of disintegration test method for ODFs [43]. For the film disintegration tests, no official guidance for the disintegration of ODF was found [44,45]. The modified pharmacopoeial disintegration test was used to close to the standard, using the basket-rack assembly specified in the USP disintegration test [19,46]. As a result of the disintegration test of the films, F5-ODF and F6-ODF showed faster disintegration times than F3-ODF when the plasticizer was added (Table 4). In the case of an orally disintegrating tablet (ODT), disintegration time is one of the essential attributes for ensuring that the ODT disintegrates within the recommended United States Pharmacopeia (USP) time of 30 s or the European Pharmacopoeia (Ph. Eur.) time of 3 min [42,47,48]. ODFs are also a solid dosage form like ODT that disintegrates rapidly upon contact with saliva in the oral cavity. Therefore, we evaluated disintegration times based on the standards of the USP and Ph. Eur. The disintegration times of all three ODFs (F3-, F5-, and F6-ODFs) complied with the USP as well as the Ph. Eur. for ODTs and F6-ODF showed the fastest disintegration time (Table 4).

### 3.3. Surface Morphology of 3D Printed ODFs

**Figure 5:**
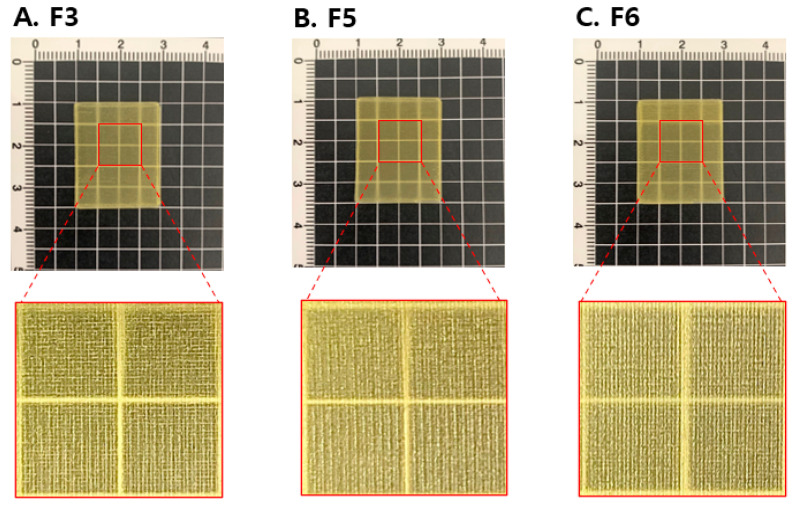
Images of the 3D printed ODFs for (**A**) F3, (**B**) F5, and (**C**) F6.

The optical images of 3D printed ODFs are shown in Figure 5. The ODFs had perpendicular shapes and a yellow color due to the OLZ content. The printed surface did not indicate a noticeable difference in the three ODFs (F3, F5, and F6) to the naked eye. The partially enlarged images showed lattice patterns printed along the XY axis without the interruption. The ODFs had clear, transparent, and homogeneous surface properties (Figure 5A–C).

**Figure 6:**
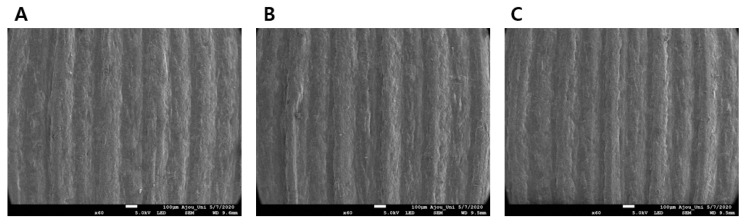
SEM images of 3D printed ODFs for (**A**) F3, (**B**) F5, and (**C**) F6.

F3-, F5-, and F6-ODFs were analyzed by SEM to investigate the topology and morphology of the final three formulations (Figure 6). All formulations had similar film formation and printing capacity without much difference between them, and the addition of different plasticizers did not affect the surface morphology of 3D printed ODFs (Figure 6B,C). The films had a soft surface with little pores. F3-, F5-, and F6-ODFs showed overall uniformity without clumps, and well-distributed extruded materials into the polymer carriers.

### 3.4. Differential Scanning Calorimetry (DSC)

**Figure 7:**
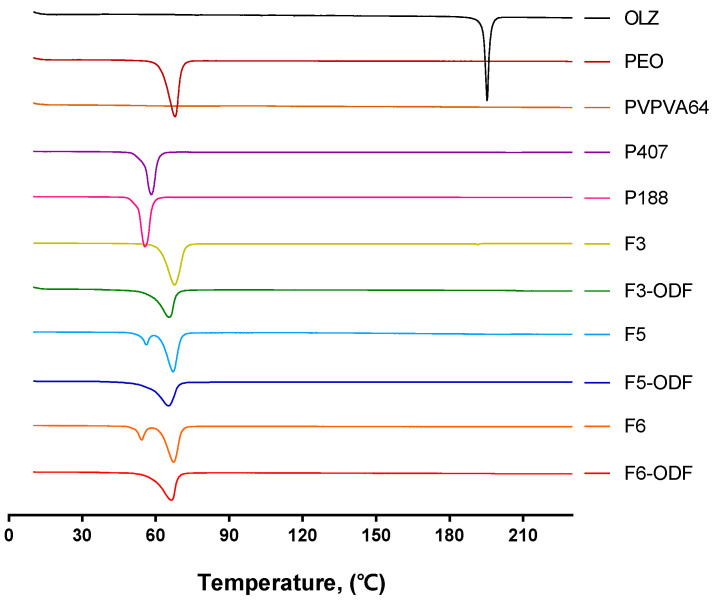
Differential scanning calorimetry (DSC) curves of free OLZ, individual polymers (PEO, PVPVA64, P407, and P188), powder mixtures before 3D printing (F3, F5, and F6), and 3D printed ODFs (F3-ODF, F5-ODF, and F6-ODF).

DSC analysis of the individual components of the powder mixtures before printing of the 3D printed ODFs was performed to investigate the thermal transition (Figure 7). As a crystalline drug, OLZ had a sharp peak and showed a melting point at approximately 195 °C. The polymers showed endothermic peaks except for PVPVA64 in the amorphous form. PEO is highly crystalline, and the DSC curve showed a melting peak at around 68 °C. The DSC curves of P407 and P188 showed an endothermic peak at 58 °C and 55 °C in accordance with their melting points.

The DSC data of formulations before printing showed endothermic peaks around the melting points of PEO, P407, and P188, which corresponded to the melting point of the polymers used. The 3D printed formulations had a relatively smooth single endothermic peak, which corresponds to the melting point of PEO. The drug peak was not detected in ODFs that had an amorphous state drug within polymeric carriers after printing.

### 3.5. Powder X-ray Diffraction (PXRD)

**Figure 8:**
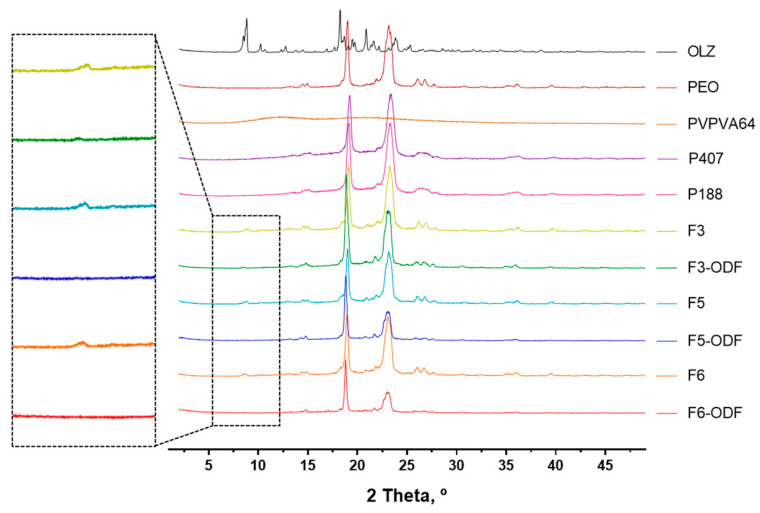
Powder X-ray diffraction (PXRD) curves of free OLZ, individual polymers (PEO, PVPVA64, P407, and P188), powder mixtures before 3D printing (F3, F5, and F6), and 3D printed ODFs (F3-ODF, F5-ODF, and F6-ODF).

X-ray diffraction analysis was performed to determine the diffraction patterns of individual components and formulations (Figure 8). The crystalline form of OLZ typically showed sharp peaks at 2θ values of 8.79° and 18.48° [49]. The PXRD peaks of the polymers used in the formulations showed characteristic peaks corresponding to PEO, P407, and P188. In contrast, PVPVA64 did not show any peaks and had wide halos indicative of the amorphous form. The physical mixtures before 3D printing showed sharp peaks that partially included crystalline peaks of OLZ at approximately 2θ = 8.79° (F3, F5, and F6 in Figure 8). 3D printed ODFs showed sharp peaks corresponding to the peaks of the polymers, but there were no OLZ crystalline peaks compared to the physical mixture (F3-ODF, F5-ODF, and F6-ODF in Figure 8). This result indicates that the drug was present in an amorphous form by HMPE in a single process of 3D printing.

### 3.6. Dissolution Test of ODFs

The apparatus and media selection are important for dissolution testing. Despite the simple orientation of the Pharmacopeia description, it is important to consider that this assay should be representative and an approach to predict the in vivo behavior [50]. However, the major methods described do not satisfactorily mimic the physiological conditions, with respect to the dissolution method conditions and apparatus [51]. Generally, the paddle apparatus (USP type II) is more widely used than the basket apparatus (USP type I) for the dissolution test of orodispersible dosage forms (e.g., orodispersible tablets, films). However, due to the limitations of both methods, many researchers have suggested the use of modified methods (e.g., dissolution media volume reduction, stirring accessory modifications, and type of dissolution medium) [51,52,53]. The primary objective of the ODFs was to disintegrate and dissolve in the mouth within a short time. To mimic the physiological conditions, the dissolution medium was modified as simulated artificial saliva [30,31,53].

**Figure 9:**
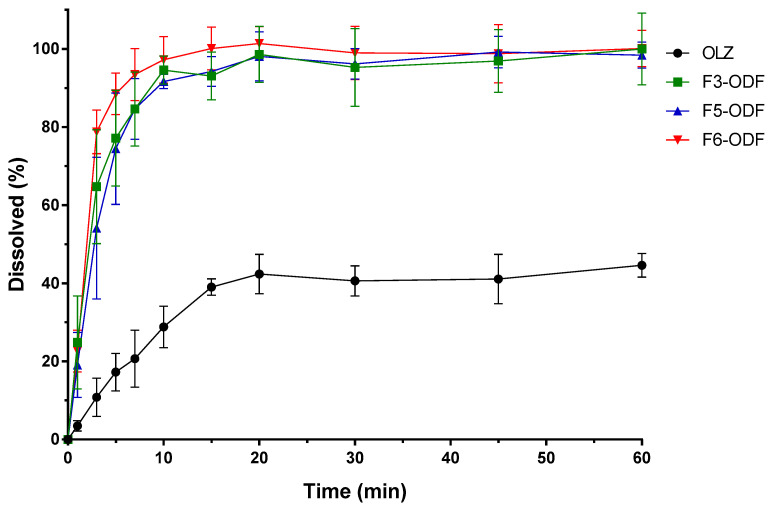
Drug dissolution profiles of free OLZ and 3D printed ODFs (F3-ODF, F5-ODF, and F6-ODF).

We evaluated the drug dissolution rates of ODFs compared with free OLZ. The drug dissolution profiles of free OLZ and the 3D printed ODFs (F3-, F5-, F6-ODFs) are shown in Figure 9. The drug dissolution for free OLZ powder and F3-, F5-, and F6-ODFs was evaluated according to the USP Apparatus II paddle method under simulated salivary conditions at 37 °C with 100 rpm of paddle rotation. All ODFs showed immediate drug dissolution profiles and an enhanced dissolution rate of OLZ compared to free OLZ powder. Free OLZ powder was only dissolved in 44.6% of the drug in 1 h, and all 3D printed ODFs dissolved more than 90% of the drug in 10min (Figure 9). The OLZ was in crystalline form in the physically mixed powder and free OLZ, and the OLZ loaded on the 3D printed ODF was converted into an amorphous form through the process of HMPE and loaded into the polymer carriers (Figure 7 and Figure 8). HME has been widely used to manufacture ASDs, and ASDs have characteristics that lead to higher API solubility and bioavailability, such as the advantages of internal free energy, saturation solubility, and maximum expansion of the surface area. Therefore, HMPE was successfully utilized for the formation of ASDs of OLZ, enhancing the solubility of poorly soluble APIs [54,55,56]. In particular, F3-ODF, which did not contain the plasticizer, and F5 with P407 showed similar dissolution patterns, reaching 84.7% and 84.6% of cumulative drug dissolution in 7 min, respectively. F6 containing P188 showed the fastest drug dissolution profile, reaching 88.5% of drug dissolution in 5 min (Figure 9). Two mathematical methods for the comparison of dissolution profiles are recommended by Moore and Flanner who describe two equations—a “difference factor” (f1) and a “similarity factor” (f2) [57,58]. Values of f1 between 0 and 15 and of f2 between 50 and 100 “ensure sameness or equivalence” of the two dissolution profiles. Both equations are endorsed by the FDA as acceptable methods for dissolution profile comparison, but the f2 equation is preferred [59]. Therefore, we calculated “similarity factor” f2 values from the dissolution profiles of the ODFs (F3, F5, and F6). As results, f2 values of F3-ODF and F5-ODF are in the range between 50 and 100 at all time points, which ensured sameness or equivalence of the two dissolution profiles of F3 and F5. However, f2 values of F3-ODF and F6-ODF are under 50 at 3, 5, and 7 min and of F5-ODF and F6-ODF are under 50 at 3–45 min, indicating that the dissolution profiles of F3-ODF and F6-ODF and of F3-ODF and F5-ODF are not equivalent to each other at early stage. Although the plasticizer was added to formulations at a small content of 5% polymeric carriers (the addition of P188 and P407), plasticizer-incorporated polymeric carriers improved the dissolution and disintegration rate of ODFs. P188 showed a better effect on the OLZ dissolution of ODF than P407. According to the standard of immediate release (IR) oral solid dosage forms by the FDA, an IR drug product is considered to be rapidly dissolving where 85% or more of the labeled amount of the drug substance is dissolved within 30 min [59]. Thus, the drug dissolution profiles of all three ODFs showed more than 80% drug dissolution in 7 min as well as 90% of drug dissolution in 10 min, which complies with the FDA guidelines for IR formulations.

The ASDs of drugs performed during the HME process could contribute to improved drug dissolution. The drug carriers consisting of hydrophilic polymers also enhanced the dissolution rates. The single step of 3D printing successfully loaded the ASDs of OLZ fabricated into ODFs by HME and enabled solubilization of OLZ, a poorly water-soluble drug.

## 4. Conclusions

The single process of 3D printing using HMPE was evaluated as an appropriate technique for the manufacture of ODFs loaded with OLZ of the amorphous form. The polymeric carriers were composed of PEO, PVPVA64, P407, and P188, and the 3D printer pneumatically extruded the molten solid dispersion and produced ODFs as the designed 3D model. All of the 3D printed ODFs with uniform drug content showed good printability, strength, and rapid disintegration time. The formulation using P188 indicated the fastest dissolution time compared to the others, and the printed films showed immediate dissolution profiles. These results are attributed to the amorphous solid dispersion of poorly water-soluble drugs as well as the composition of hydrophilic polymers, and it was prepared by a single process without further processing.

This study uses the technique of immediate manufacturing without the complex processing and handling of conventional 3D printing, and ASDs in 3D printed formulations are prepared by HMPE. The single process of 3D printing applies to the immediate manufacture of drugs containing various dosage forms and amounts in pharmaceutical studies.

## Figures and Tables

**Table 1:** Optimized printing parameters via pre-screening.

Slicer Settings	Set Values and Type
Printing Speed (mm/s)	4
Moving Speed (mm/s)	6
Infill Pattern	Line
Filling Density (%)	100
Bed Temperature (°C)	35

**Table 2:** Composition of placebo for polymeric carriers (% *w/w*).

Placebo	PEO	PVPVA64	Printing Temperature (°C)	Film Forming Capacity
P1	100	0	100	++
P2	90	10	125–130	++
P3	80	20	135–140	++
P4	70	30	145–150	++
P5	60	40	150–155	+
P6	50	50	155	−

(−) No extrusion, (+) unstable film, (++) good extrusion, and film formation [39].

**Table 3:** Composition of the orodispersible film (ODF) formulations (% *w/w*).

Formulation	OLZ	PEO	PVPVA64	P407	P188	Printing Temperature (°C)
F1	5	95	-	-	-	170
F2	5	85	10	-	-	165
F3	5	75	20	-	-	160
F4	5	65	30	-	-	160
F5	5	70	20	5	-	160
F6	5	70	20	-	5	160

(-): Not added.

**Table 4:** Characterization of the final formulations for ODFs containing 5 mg OLZ.

Formulation	Mean Mass ± SD (mg)	Drug Loading ± SD (%)	Thickness ± SD (μm)	Strength ± SD (N)	Disintegration Time ± SD (s)
F3	102.9 ± 3.3	98.0 ± 2.4	243.3 ± 5.7	14.3 ± 0.2	21.7 ± 1.5
F5	103.2 ± 5.5	98.5 ± 1.5	242.3 ± 7.1	10.8 ± 2.7	17.7 ± 1.5 ^b^
F6	102.9 ± 2.4	99.6 ± 2.1	243.3 ± 11.5	15.6 ± 0.6 ^a^	17.0 ± 1.7 ^b^

^a^*p* < 0.05 significantly different from F5; ^b^ *p* < 0.05 significantly different from F3.
